# UNDERSTANDING AND ENDING AGEISM: AN INNOVATIVE COLLEGE COURSE

**DOI:** 10.1093/geroni/igae098.2944

**Published:** 2024-12-31

**Authors:** Jennifer Sasser

**Affiliations:** Portland Community College, PORTLAND, Oregon, United States

## Abstract

Understanding and Ending Ageism is an online community college course developed as part of a grant-funded project that aimed to raise awareness about aging and ageism, as well as to make the case for integrating these dimensions of human experience into college-wide diversity, equity, and inclusion initiatives. Designed and first offered in 2016, the course continues to be offered two terms per year and has been periodically refreshed to incorporate new perspectives and resources (including the Reframing Aging Initiative and updated AGHE Competencies). Course content includes a comprehensive overview of the characteristics, mechanisms and effects of ageism (and how ageism is experienced across the life course and at different ages and stages); examination of how ageism intersects with other “isms” in forming a system of oppression and yet is not often enough considered in discussions about diversity, equity, and inclusion; identification and planning of interventions and projects connected to increasing age-appreciation and challenging ageism; themes of aging-awareness, social justice and intergenerational solidarity; and an emphasis on reflection, critical thinking, and praxis. Unique features of the course include its collaborative learning design and its focus on “practices” that we can engage in at every level at which ageism operates and at which we can intervene: the individual; interpersonal; social/organizational; societal/political; and cultural. In addition to weekly assigned readings and roundtable discussions, students create an Ending Ageism Synthesis & Action Project as their capstone assignment.

